# A self-management intervention for bipolar disorder using a WeChat mini program (Mood Calm): study protocol for intervention design, development, and a preliminary trial

**DOI:** 10.3389/fpsyg.2026.1784369

**Published:** 2026-06-19

**Authors:** Xiaoling Lin, Zhengling Ba, Dali Lu, Ruoyun Ma, Minhua Chen, Caihong Zhu, Yingtao Liao, Xueying Yu, Yujing Liu, Duoduo Lin

**Affiliations:** 1 School of Nursing, Xiamen Medical College, Xiamen, China; 2 School of Nursing, Sun Yat-sen University, Guangzhou, China; 3 Department of Psychiatry, Xiamen Fifth Hospital, Xiamen, China; 4 Department of Cardiology, China-Japan Friendship Hospital, Beijing, China; 5 Department of Psychiatry, The Third Affiliated Hospital of Sun Yat-sen University, Guangzhou, China; 6 Sun Yat-sen University Cancer Center, Guangzhou, China; 7 Xiamen Xianyue Hospital, Xianyue Hospital Affiliated with Xiamen Medical College, Fujian Psychiatric Center, Fujian Clinical Research Center for Mental Disorders, Xiamen, China

**Keywords:** bipolar disorder, feasibility, Mood Calm, self-management, smartphone

## Abstract

**Introduction:**

Bipolar disorder (BD) is a severe, chronic illness characterized by frequent relapses and persistent interepisode symptoms, even with standard pharmacological treatment. Digital self-management tools have the potential to broaden the reach of evidence-based approaches and improve healthcare delivery through immediate evaluation, tailored guidance, and notifications to healthcare providers, while collecting self-report and behavioral data to guide treatment decisions. This study describes the design, development, and a preliminary evaluation of “Mood Calm”, a comprehensive self-management intervention for BD delivered via a WeChat mini-program.

**Methods:**

The intervention protocol was developed through a multiphase process. First, guided by Cognitive Behavioral Theory, an initial protocol was constructed integrating six modules: symptom monitoring, medication management, crisis identification, healthy lifestyle support, psychoeducation, and cognitive functioning training. Plans for wearable device integration were also included to enable passive monitoring of sleep and activity. Second, a literature review identified core functional components of digital self-management interventions for BD, and the protocol was subsequently refined via a Delphi expert panel. Third, the “Mood Calm” mini-program was developed using an agile framework. Then, a four-week, single-arm preliminary trial was conducted with five remitted patients with BD to assess feasibility and usability of the digital self-management modules delivered through the “Mood Calm” mini-program. These modules included symptom monitoring, medication management, lifestyle tracking via wearable device, and cognitive functioning training, but excluded psychoeducation and crisis intervention components which are planned for future randomized controlled trial (RCT).

**Results:**

All participants completed the trial, reporting high satisfaction and describing the application as user-friendly and easy to operate. However, system data indicated suboptimal self-reported medication adherence for three participants, prompting an immediate design revision to implement a one-click reporting function.

**Discussion:**

Findings support the feasibility and acceptability of the “Mood Calm” mini-program as a potential digital adjunct for BD self-management in China. This study establishes a protocol for a comprehensive intervention and provides preliminary evidence for the mini-program-based components. Future research will evaluate the full intervention, incorporating group psychoeducation sessions, in a definitive RCT.

## 1 Background

Bipolar disorder (BD) is a chronic, recurrent condition affecting approximately 2% of the global population, characterized by episodic fluctuations between depressive and manic states, that are often accompanied by interepisodic subsyndromal symptoms (Grande et al., 2016; McIntyre et al., 2020; Oliva et al., 2024). BD is associated with high mortality, impairment in psychosocial functioning, cognitive performance, and quality of life, and it reduces life expectancy (Catalá-López et al., 2013; GBD 2019 Mental Disorders Collaborators, 2022; Kessing et al., 2015). Frequently, individuals with BD lack insight into their diagnosis and symptoms, particularly during manic phases, which leads to poor prognosis (Vieta and Valentí, 2013). Although pharmacotherapy remains the primary treatment for BD, high rates of recurrence, interepisode symptoms, and psychosocial impairment often persist even after effective medication management (Geddes and Miklowitz, 2013; Grande et al., 2016; Grunze et al., 2013; Oliva et al., 2024).

Adjunctive psychological interventions, when combined with pharmacological treatment, have demonstrated efficacy in reducing episode recurrence and symptom severity, as well as enhancing quality of life (Bond and Anderson, 2015; Miklowitz et al., 2021; Reinares et al., 2014). Current clinical guidelines recommend integrating adjunctive psychotherapy, highlighting the necessity of continuous mood symptom monitoring and the implementation of recovery-focused interventions (Fountoulakis et al., 2017; Goodwin et al., 2016; Yatham et al., 2018). However, several barriers significantly restrict the widespread implementation of psychosocial interventions, such as limited geographical and financial resources, service capacity constraints, perceived inefficacy, self-stigma, lack of insight, and low service uptake (Brown et al., 2015; Colom, 2011; Michalak et al., 2016; Nicholas et al., 2017; Reinares et al., 2014; Stafford and Colom, 2013). As such, there is a pressing need to establish accessible, scalable, and efficient models for delivering psychosocial support to a broader spectrum of BD patients.

Self-management interventions, especially those facilitated through digital health platforms, represent a compelling strategy; by reducing dependence on healthcare providers, they can empower individuals, provide a greater sense of agency, and overcome traditional access barriers (Michalak et al., 2016; Nicholas et al., 2017). These strategies have proven effective in improving psychosocial functioning, quality of life, and medication adherence, as well as in reducing relapse risk in patients with BD (Michalak et al., 2019; van den Heuvel et al., 2019). Internet-based resources can enhance the accessibility and affordability of self-management approaches (Hidalgo-Mazzei et al., 2015a), a particularly salient advantage given the prevalent use of the internet among patients with BD seeking health information (Bauer et al., 2016).

The rapid proliferation of smartphones has further spurred research into mobile health solutions for chronic conditions like BD (Bauer et al., 2016). Smartphone-based tools are often perceived by patients as comfortable, user-friendly, and non-invasive methods for self-management (Bush et al., 2013). They enable real-time monitoring of symptoms (Faurholt-Jepsen et al., 2013; Morton et al., 2025; Stanislaus et al., 2021) and can deliver tailored psychoeducational content based on individual data (Ben-Zeev et al., 2013). Supporting this, studies report high acceptability and satisfaction with specific applications. For instance, a 12-month trial of the PHR-BD applications found that 84.6% of participants with BD continued to use and would recommend it, citing improved insight and perceived control over symptoms, though a high attrition rate was a limitation (van den Heuvel et al., 2018). Hidalgo-Mazzei et al. (2016) reported that 82% of patients with BD considered the SIMPLe smartphone applications to be effective and appropriate for self-management, with overall satisfaction reaching 86%. Notably, the SIMPLe accurately identified suicide risk in 60% of cases, with false alarms mainly resulting from system testing. Research also indicates patient preference for features like mood diaries, visual feedback, and objective monitoring of sleep and physiology to enhance engagement (Daus et al., 2018), and supports the role of smartphones in remote daily mood monitoring (Stanislaus et al., 2021). Despite the demonstrated promise of digital tools, research on BD self-management applications remains scarce in China.

Notably, the integration of wearable devices with mobile applications has shown potential for passive monitoring of sleep, physical activity, and heart rate, thereby reducing self-reporting burden (Cochran et al., 2018; Van Til et al., 2020). Approximately 72.3% of patients expressed willingness to use wearables, with sleep monitoring identified as particularly valuable for increasing self-awareness and self-management consciousness. However, forgetting to upload data remains an adherence barrier (Cochran et al., 2018; Van Til et al., 2020). Beyond feasibility, meta-analyses confirm that wearable devices can remotely monitor physiological markers such as sleep, activity, and heart rate, as well as track emotional fluctuations in real-time to assist patients with symptom management (Javelot et al., 2014). Additionally, wearables offer the advantages of being intelligent, non-invasive, and discreet, which can help alleviate stigma associated with mental illness (Ben-Zeev and Depp, 2012). These features suggest that a combined wearable and mobile applications approach, especially with integrated reminders to support adherence, could be a valuable modality for supporting self-management in individuals with BD.

Among available mobile intervention platforms, WeChat mini-program, which integrated within China's most widely-used social media applications, are at the forefront due to their low costs of development, promotion, and maintenance (Ma and Wang, 2022). These platforms deliver app-like functionality without requiring separate downloads, thereby conserving device storage while maintaining accessibility and convenience (Liu et al., 2023). Thus, the primary aim of this study is to design, develop, and conduct a preliminary evaluation of a self-management intervention for BD delivered via a WeChat mini-program (*Mood Calm*).

## 2 Intervention development

**Figure 1:**
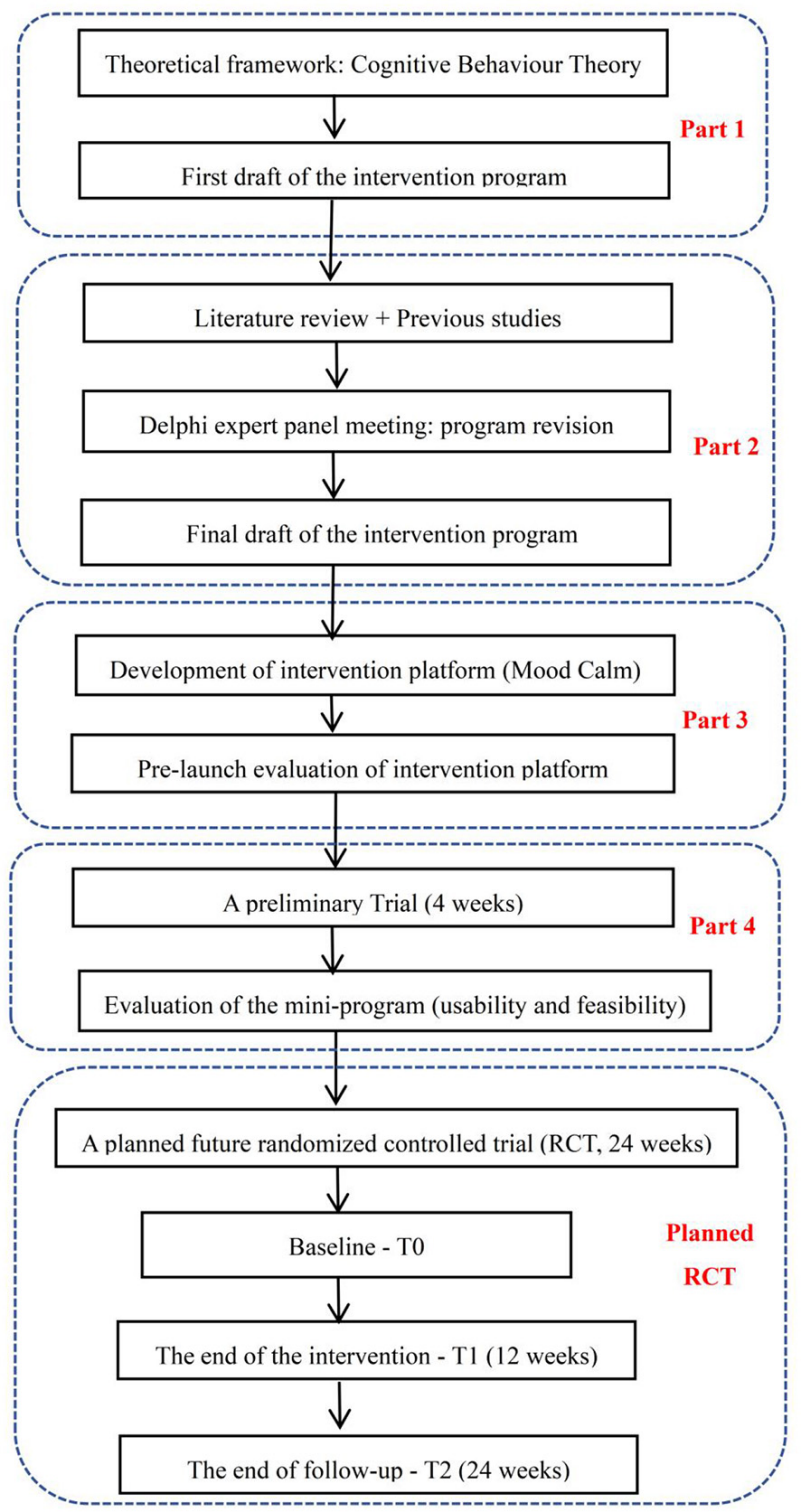
Flowchart of the study.

The development of the “Mood Calm” intervention followed a systematic, multiphase process (see Figure 1). This section describes the theoretical framework and the initial program development.

### 2.1 Theoretical framework

In this study, we adopted Cognitive Behavior Theory (CBT) as the theoretical foundation for developing the intervention program. CBT is a structured psychotherapeutic approach, formed through the integration and mutual supplementation of cognitive theory and behavioral therapy, based on the cognitive therapy techniques established by American psychologist Beck in 1976 (Beck, 1997). It has become one of the core pillars of contemporary international psychotherapy (Beck, 1997; Hofmann et al., 2013). Cognitive theory posits that cognitive processes, together with an individual's emotions and behaviors, determine psychological outcomes, and that individuals can modify their thoughts by altering cognitive processes, thereby correcting emotions and behaviors. Behavioral therapy contends that behaviors are learned and can be restrained, corrected, or replaced through practical interventions.

CBT represents the synthesis of these two approaches, asserting that cognitive processes determine behavior, while behavioral changes can also influence cognition. CBT employs corrective techniques to amend irrational beliefs, continuously linking cognitive and behavioral modifications to establish a positive feedback loop that replaces previously negative cycles, thereby alleviating or eliminating maladaptive symptoms (Dattilio, 2000). The core concept of CBT encompasses a set of psychotherapeutic methods aimed at improving emotional and behavioral issues and enhancing mental health by altering maladaptive thinking and behavioral patterns (Dattilio, 2000).

**Figure 2:**
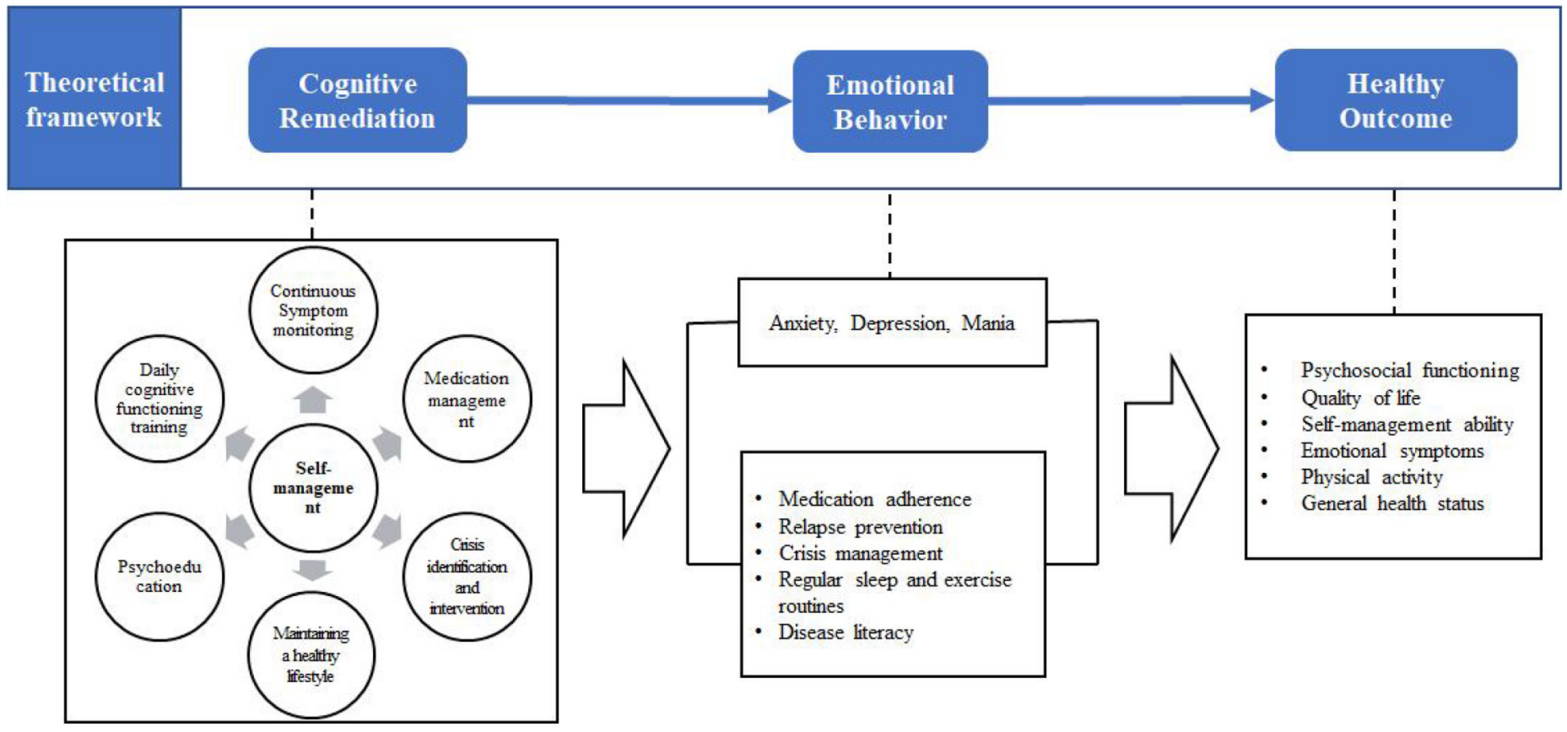
Theoretical framework.

The conceptual framework of this study was constructed based on the above theory. The self-management intervention in this research includes six modules: continuous symptom monitoring, medication management, crisis identification and intervention, maintaining of a healthy lifestyle, psychoeducation, and daily cognitive functioning training. These six components of the self-management intervention are designed to improve the cognitive processes of patients with BD, thereby ameliorating negative emotions and behaviors, enhancing disease self-management ability and medication adherence, improving sleep and exercise patterns, and ultimately improving functional outcomes and quality of life (see Figure 2).

### 2.2 Initial intervention protocol

Based on the above framework, the research team conducted group discussions to develop the preliminary program for the self-management intervention protocol. The complete intervention, planned for a future randomized controlled trial (RCT; see Figure 1), consists of six key modules, as detailed below. The intervention services and evaluation and feedback schedule for each functional module are detailed in Table 1.

**Table 1:** Preliminary functional modules.

Functional module	Methods	Contents	Evaluation and feedback
Continuous symptom monitoring	① Mood calm ② Wearable devices ③ WeChat	Self-assessment, identification, and management of symptoms, include depression, anxiety, and suicidal ideation.	Weekly feedback
Medication management	① Mood calm ② Tencent meeting ③ WeChat	① Each patient required to record their medication intake daily. ② Reminder via SMS or WeChat (or by phone if necessary) will be issued when medication noncompliance.	Daily feedback
Crisis identification and intervention	① Mood calm ②Tencent meeting ③ Phone ④ Face to face (*if necessary*)	Recognition of prodromal indicators of recurrent depressive episodes and suicidal behavior, and timely proactive interventions.	As determined by patient feedback
Maintaining a healthy lifestyle	① Mood calm ② Wearable devices ③ WeChat	① Sleep monitoring: duration, deep sleep phases, frequency, and duration of wakefulness episodes. ② Nutritional guidance: provision of evidence-based information on healthy dietary practices. ③ Physical activity tracking: daily step count and energy expenditure analysis.	Weekly feedback
Psychoeducation	① Mood calm ② WeChat ③ Tencent meeting ④ Face to face (if necessary)	① Group psychoeducation consist of six sessions, conducted biweekly, each lasting 60 min. ② Timely delivery of information to patients and/or their family members. ③ Individualized psychoeducation can be provided as needed.	Twice weekly feedback
Daily cognitive functioning training	① Mood calm ② WeChat	① Deliver information to patients regarding cognitive functioning and relevant techniques, such as videos and images.	Twice weekly feedback

#### 2.2.1 Continuous symptom monitoring

**Figure 3:**
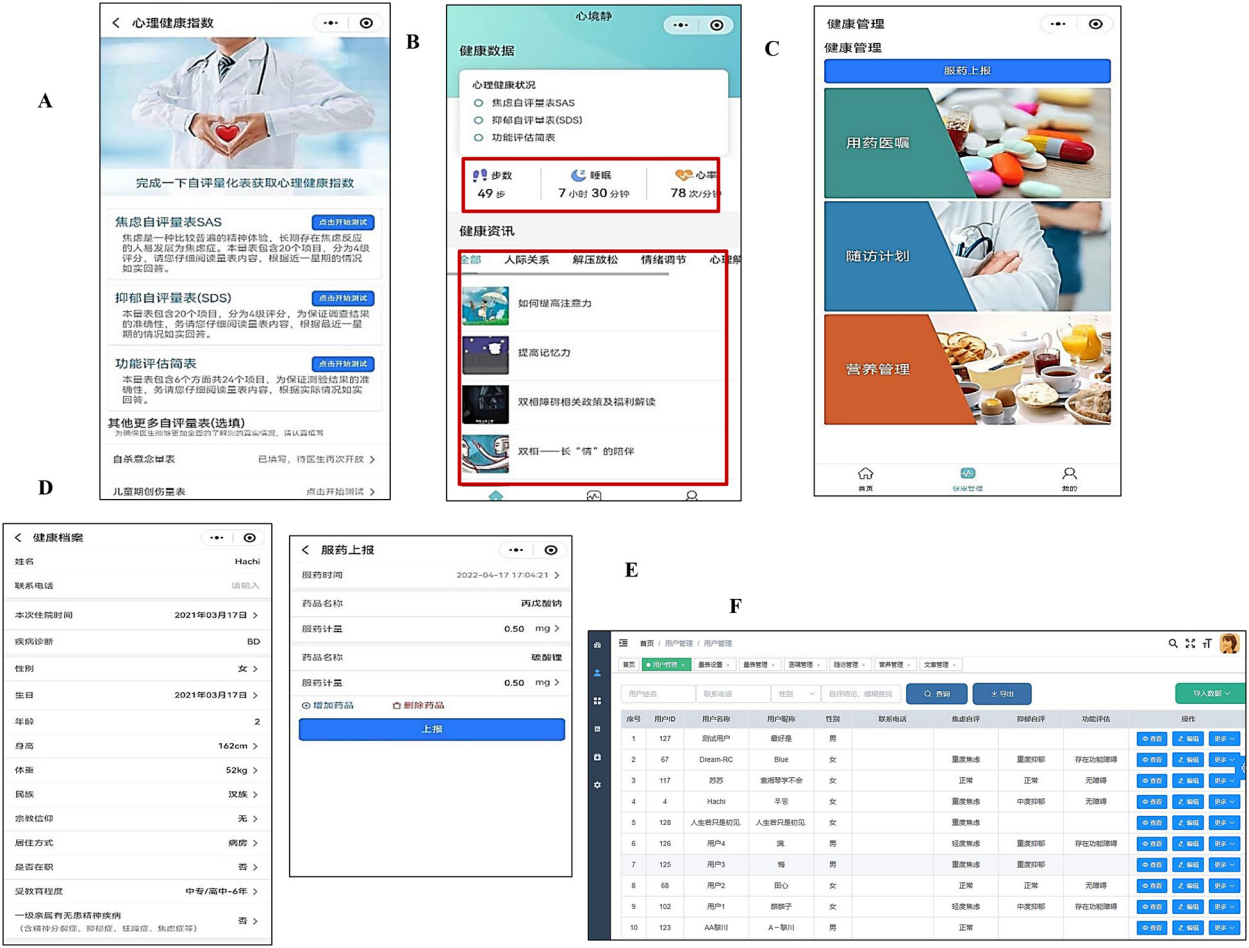
The WeChat mini-program “Mood Calm” modules and functions. **(A)** Psychological self-assessment; **(B)** monitoring of physical activity, sleep, heart rate, and healthy information; **(C)** healthy management; **(D)** daily self-report medication; **(E)** personal center; **(F)** healthcare provider-side.

This module primarily consists of three components: self-assessment, identification, and management of symptoms. This module provides self-assessment scales for emotions such as depression, anxiety and suicidal ideation (see Figure 3). Patients can access and complete these scales by clicking on them, and the system will provide emotional guidance based on an integrated scoring algorithm to assist patients in recognizing their emotional symptoms. Hypo/manic symptoms will be assessed by research staff using the Young Mania Rating Scale (YMRS; Young et al., 1978) at baseline (*T*0), at the end of the intervention phase at 12 weeks (*T*1), and at the end of the follow-up at 24 weeks (*T*2). Although this scale is not integrated into the daily self-monitoring module due to its clinician-administered nature, it remains a core outcome measure for evaluating the full spectrum of mood symptoms.

#### 2.2.2 Medication management

This module is designed to remind and monitor patients regarding their medication adherence, ultimately enhancing treatment compliance (see Figure 3). Medication regimens can be customized for each patient based on their specific needs, including drug names, dosages, and administration times for daily medications. Patients are required to record their medication intake daily; these data are transmitted to the healthcare providers' data platform and serve as key evidence when determining the necessity of medication interventions. If a patient fails to record their medication for more than 1 day, healthcare professionals will issue a reminder via SMS or WeChat (*or by phone if necessary*). If the patient has not entered any records for over a week, healthcare professionals will follow up with a phone call to further assess the situation and implement appropriate measures.

#### 2.2.3 Crisis identification and intervention

Crisis management involves formulating strategic protocols aimed at preventing disease recurrence, specifically targeting the return of depressive episodes and suicidal behavior. Data collected from the WeChat “Mood Calm” mini-program, including patient-reported mood assessment scores, medication adherence rates, and activity metrics, are utilized. Clinicians can then conduct preliminary evaluations to detect prodromal symptoms indicative of relapse. Upon identification of early warning signs, research personnel leverage back-end analytics to conduct continuous real-time monitoring of the patients' status, collaborate with family members to reinforce support mechanisms, and implement proactive interventions to mitigate relapse risk.

#### 2.2.4 Maintaining a healthy lifestyle

This module addresses lifestyle factors associated with BD, including the maintenance of regular and sufficient sleep patterns, healthy dietary habits (*such as consistent meal schedules and monitoring the intake of stimulants like caffeine and sugar*), and regular physical activity. Monitoring in this module is supported by wearable devices (Lexin Smart Band, see Figure 3).

First, sleep patterns are monitored via the Lexin Smart Band (*model: Lexin M5*). According to WHO recommendations, the study uses a duration of 6.0–7.5 h per night as the standard; if a patient falls short of this standard for 1 consecutive week, healthcare professionals will use the wristband data to remind them to prioritize sleep and avoid staying up late.

Second, patients are also encouraged to maintain a regular eating schedule and monitor the intake of stimulants such as caffeine and sugar. The WeChat mini-program provides educational resources on healthy eating and informs patients that excessive consumption of caffeine and sugar can cause emotional fluctuations.

Third, activity levels are also monitored via the Lexin Smart Band. Exercise physiology research indicates that adults need to expend at least 300 kcal of energy per day to maintain a healthy status. The wristband automatically converts step count to calories burned, allowing researchers to assess whether daily exercise targets are met. If activity levels fall short for a full week, researchers will remind patients to increase physical activity and offer personalized exercise recommendations. Upon enrollment, patients will receive the wristband and training on its use, involving both the patient and their family members. Patients and caregivers are clearly instructed that the wristband should be worn on the non-dominant wrist to monitor daily activity and sleep data. Except when charging, patients are expected to wear the device continuously to ensure data completeness. Every Sunday, either the patient or family member must synchronize the collected data to the cloud.

#### 2.2.5 Psychoeducation

Group psychoeducation sessions will be conducted online through Tencent Meeting, a videoconferencing platform, with one nurse assigned to each group and each group consisting of 8 to 10 patients and/or caregivers. Sessions will be held once every 2 weeks, with each session lasting 60 min, for a total of six sessions. The program will emphasize the stress-vulnerability model of BD and the pivotal role of family support. Key topics will include the etiology, symptoms, diagnosis, treatment, adverse effects, functional outcomes, self-management, prognosis and relapse, the significance of family support and its implementation, as well as communication skills between family members and patients. Patients will be encouraged to recognize and manage their own emotional symptoms to alleviate subthreshold depressive symptoms, enhance treatment adherence, and improve social support.

The specific content of the six sessions is as follows: (i) Understanding BD; (ii) Pharmacological treatment, side effects, and adherence; (iii) Self-management strategies; (iv) Disease prognosis, rehabilitation, and psychosocial factors; (v) Family-centered care, focusing on marriage, childbirth, and legal issues; and (vi) Policy interpretation, legal considerations, and welfare resources related to the management of severe mental illnesses.

On the day each session concludes, research assistants will distribute relevant educational materials via a WeChat mini-program and the public account “Mood Calm” for patients and their families to review and study. Additionally, individualized psychoeducation will be provided in person when patients attend hospital visits for assessments (see Figure 3).

#### 2.2.6 Daily cognitive functioning training

This module is designed to provide patients with information regarding the concepts, definitions, and daily enhancement strategies related to cognitive functioning and relevant techniques. It aims to encourage patients to utilize various cognitive enhancement methods in their everyday lives. The topics covered include: (i) Understanding attention, strategies for improvement and practical applications in daily life; (ii) Understanding memory, techniques for enhancing memory in everyday activities; (iii) Leveraging reading to improve memory and undertake reading-based tasks; (iv) Understanding executive functions, strategies for enhancement and their implementation in routine situations.

## 3 Literature review and expert panel

### 3.1 Literature review of mobile applications for BD self-management

#### 3.1.1 Data sources and search strategy

The study was reported according to the PRISMA (*Preferred Reporting Items for Systematic Reviews and Meta-Analyses*) guideline extension for reporting scoping reviews (Page et al., 2021). The review protocol was not registered with a database. Citations were retrieved from four English databases (*PubMed, Web of Science, Embase*, and *PsycINFO*) and four Chinese databases (*CNKI, CBM, WFSD*, and *CQVIP*), using search terms grouped by key aspects. For the Chinese databases, search terms such as “双相障碍,” “自我管理,” “移动应用,” “智能手机,” “移动网络,” and “手机应用” were used. For the English databases, the keywords were “bipolar disorder” “mobile applications,” and “self-management.” Review articles published from September 2012 to September 2022 were retained, including studies published in either English or Chinese. Although studies published after this date are not included, the 12 eligible articles identified sufficiently informed the core functional components of our intervention. Ongoing literature monitoring will be conducted to inform future trial updates.

Earlier articles were excluded for several reasons: (i) technological changes; (ii) the need to balance comprehensiveness of the search with the manageability of the volume of articles; and (iii) the inclusion of articles that incorporated studies published before 2012, which allowed us to capture relevant earlier work.

#### 3.1.2 Study selection

Titles and abstracts of all retrieved articles were independently screened by two members of the research team trained in literature review methods (*BZ* and *MY, YX*, or *LY*), while full-text articles were independently screened for inclusion by two members of the research team (*BZ* and *MY*, or *YX*). The final selection was made based on the eligibility criteria outlined above. Any disagreements between the reviewers were resolved by LX. Any disagreements regarding inclusion were resolved through discussion or consultation with a third reviewer.

#### 3.1.3 Inclusion and exclusion criteria

Studies were included if they met the following criteria: (i) focused on self-management among patients with BD; (ii) documented the use or development of self-management interventions through mobile health technologies; (iii) provided specific details regarding the functional modules. Exclusion criteria were as follows: (i) publications not in English or Chinese; (ii) duplicate articles; (iii) studies without available full texts, and (iv) non-original research articles.

#### 3.1.4 Synthesis

A total of 125 articles related to self-management in BD were initially identified. After excluding non-Chinese and non-English papers, duplicate studies, articles not meeting inclusion criteria, and publications with inaccessible full texts, 12 studies were included. Key information on applications supporting self-management in BD was extracted and summarized, with a focus on disease self-management interventions. In this study, the relevant literature was categorized using the following criteria: author, publication year, country, mobile application name, main features and functions, research participants and sample size, study duration, assessment methods, outcome measurement time points, outcome indicators, and the tools used for assessment. Detailed information is presented in Table 2.

**Table 2:** Summary of construction and evaluation methods of mobile applications related to bipolar disorder (*n* = 12).

References/ Country	Mobile app name	Main content and functions	Target population	Study duration	Evaluation method	Outcome measurement time points	Outcome indicators and measurement tools
Jonathan et al. (2021)/USA	LiveWell	① Basic modules: diet, exercise plans. ② Toolbox: Self-assessment surveys and skill exercises. ③ Health plans. ④ Daily check-in: monitoring medication use, sleep duration. ⑤ Daily review: task completion. ⑥ Cognitive behavioral therapy-based interactive elements.	BD patients (*n* = 11)	8 weeks (pilot study)	RCT	Daily login to report information	① Early warning symptoms: health rating scale. ② Usability testing: interview + custom task completion scale + pilot study exit questionnaire.
Pozza et al. (2020)/Italy	Bip.App	① Disease awareness. ② Medication adherence. ③ Early identification of prodromal and relapse symptoms, regularity of lifestyle.	BD patients	Ongoing	Multi-center RCT	Baseline, 4th month, 8th month, and 1 year	① Severity of depression and mania: HAMD and YMRS. ② Medication adherence: blood drug concentration and self-monitoring diary. ③ Psychotic symptoms: positive and negative syndrome scale. ④ General health status: health status questionnaire. ⑤ Psychosocial function: global assessment of functioning scale. ⑥ App usage satisfaction: satisfaction questionnaire.
Van Til et al. (2020)/USA	Lorevimo	① Recording manic and depressive symptoms and medication adherence. ②Symptom reports. ③ Visualizing patient symptoms and activity data (by Fitbit) including sleep, exercise, and heart rate. ④ Reminder function.	BD patients (*n* = 47)	6 weeks	Two-arm RCT	Weekly	① Severity of depression and mania: HAMD and YMRS. ② General health status: health status questionnaire. ③ Sleep, exercise, and heart rate: activity tracker.
van den Heuvel et al. (2018)/Netherlands	PHR-BD	① Standard modules: medical records, medications, treatments, and passports. ② Additional modules: (a) General disease information. (b) Laboratory results and reports. (c) Personal information. (d) Mood charts. (e) Crisis plans. ③ Reminder function.	BD patients (*n* = 66) and healthcare providers (*n* = 11)	12 months	Pre-post self-controlled trial	Baseline, 12th month	① Severity of depression and mania: self-rated depression scale and self-rated mania scale. ② User experience: self-made questionnaire. ③ General psychological function: 45-item outcome questionnaire. ④ Quality of life: Manchester short assessment of quality of life.
Kuzman et al. (2017, 2018)/Croatia	PsyLOG	① My drug effects: side effect selection. ② My lifestyle: recording smoking, alcohol, exercise, etc. ③ My charts: visualizing side effect severity and lifestyle changes. ④ My medications: type and dosage. ⑤ My strategies. ⑥ My supporters.	Schizophrenia and BD patients (*n* = 78)	6 months	RCT	Baseline, 1st month, 3rd month, and 6th month	① Medication side effects: Glasgow antipsychotic side-effect scale. ② Biochemical tests: blood glucose and lipid measurement. ③ Body mass index.
Schwartz et al. (2016)/USA	EMA	Monitoring mood, energy, thought speed, impulsive behavior, and social stress.	BD patients (*n* = 20)	2 weeks	RCT	① Visual analog scale: daily feedback. ② Satisfaction: at study completion.	① Mood, energy, thought speed, and impulsive behavior: visual analog scale. ② Social stress: custom stress scale. ③ Satisfaction feedback: qualitative interview.
Beiwinkel et al. (2016)/Germany	SIMBA	① Reporting mood states. ② Monitoring physical activity. ③ Monitoring social activities.	BD patients (*n* = 14)	12 months	Prospective observational study	① Mood feedback: daily. ② Depression and mania symptoms: every 8 weeks.	① Severity of depression and mania: HAMD and YMRS. ② Physical activity: mobile phone sensors. ③ Social interactions: number and duration of post-discharge phone follow-ups.
Hidalgo-Mazzei et al. (2015b, 2016)/Spain	SIMPLe	① Daily tests: mood, sleep duration, medication adherence, etc. ② Daily mood feedback. ③ Weekly tests: suicide risk assessment. ④ Daily mental health education messages. ⑤ Suicide reminder function.	BD patients (*n* = 51)	3 months	Pre-post self-controlled trial	Baseline, 3rd month	① Severity of depression and mania: HAMD and YMRS. ② Functional level: functional assessment short test. ③ Medication adherence: medication adherence rating scale.
Lauder et al. (2015)/Australia	Moodswings (moodswings-plus)	① Basic functions: monitoring mood and activity, assessing prodromal symptoms, preventing relapse, setting SMART goals. ② Supplementary functions—cognitive behavioral therapy: life chart compilation, cognitive strategies: self-reflection, problem-solving, identifying personal triggers, relapse prevention planning, etc.	BD patients (*n* = 156)	12 months	RCT	① Relapse and medication adherence: 10th week, 3rd month, 6th month, and 12th month. ② Others: baseline, 3rd month, 6th month, and 12th month.	① Severity of depression and mania: Self-rated mania scale and Montgomery-Åsberg depression rating scale. ② Relapse: self-report questionnaire and phone interview. ③ Social support: social support scale. ④ Medication adherence: medication adherence rating scale.
Murray et al. (2015)/Australia	ORBIT	① Introduction: intervention purpose, precautions. ② Self-acceptance: developing self-acceptance and self-compassion amidst persistent symptoms. ③ Mindfulness: regulating emotions and improving sleep through self-awareness. ④ Values and goals: identifying personal values, setting meaningful goals.	BD patients (*n* = 26)	3 weeks	Pre-post self-controlled trial	Baseline, 3rd week	① Quality of life: brief quality of life in bipolar disorder scale. ② Levels of depression, anxiety, and stress: depression-anxiety-stress scales.
Depp et al. (2015)/USA	PRISM	① Mood feedback. ② Mental health education. ③ Early warning of symptoms, triggering factors, and coping strategies.	BD patients (*n* = 104)	24 weeks	RCT	Baseline, 6th week (midpoint), 12th week (post-treatment), and 24th week (follow-up)	① Severity of depression and mania: Montgomery-Åsberg depression rating scale and YMRS. ② Functional impairment: disability in daily life assessment scale.
Wenze et al. (2014)/USA	IABD	① Treatment adherence intervention based on ecological momentary intervention. ② Reminder function.	BD patients (*n* = 14)	2 weeks (pilot study)	RCT	① Depression and mania measurement: Baseline and post-intervention. ② Satisfaction and perceived helpfulness: post-intervention interview.	① Severity of depression and mania: rapid depression symptom scale and self-rated mania scale. ② Overall satisfaction and perceived helpfulness with IABD: custom 5-point rating scale.

Bip.App, bipolar mobile application; PHR-BD, personal health record-bipolar disorder; EMA, ecological momentary assessment; SIMBA, social information monitoring for patients with bipolar affective disorder; ORBIT, online, recovery-focused, bipolar individual therapy; PRISM, personalized real-time intervention for stabilizing mood; IABD, improving adherence in bipolar disorder; RCT, Randomized Controlled Trial.

#### 3.1.5 Summary of overall findings

Based on a review of the literature, the key components of intervention design in this category of research are summarized as follows.

**Functional modules:** Synthesizing the functional modules incorporated within the mobile applications included in the studies, nine primary categories emerge: symptom monitoring, medication management, psychoeducational information delivery, cognitive training, crisis identification and intervention, relapse prevention, behavioral monitoring, general health status tracking, and individual record keeping.

**Implementation methods:** The primary mode of intervention in these studies is through the use of mobile applications. Some studies also leverage wearable devices, such as smartphone sensors and activity trackers, to monitor variables including physical activity, sleep patterns, and heart rate.

**Intervention period:** Generally, pilot interventions range from 2 to 12 weeks, while formal intervention periods typically span 10–24 weeks. The shortest reported intervention lasted 6 weeks, with the longest extending up to 12 months. The shortest follow-up period was 12 weeks, and the longest follow-up extended 9 months beyond the conclusion of the intervention.

**Outcome measurement:** Methods of Measurement combine online and offline approaches to assess outcomes. Online assessments include the completion of relevant questionnaires via the mobile applications and follow-up by telephone; when necessary, data are collected using wearable devices such as smartphone sensors and activity trackers. Offline assessments generally involve semi-structured interviews to evaluate patient status. Regarding types of outcome measures, due to the lack of self-management scales specifically tailored for individuals with BD, outcome indicators vary across studies. Given the multifaceted nature of self-management, researchers typically select outcome variables that closely align with the functions of the mobile applications, such as emotional symptoms (e.g., *anxiety, depression*, and *mania*), psychosocial functioning, quality of life, medication adherence, relapse rates, perceived social support, physical activity, and general health status (including *sleep, exercise*, and *heart rate*).

### 3.2 Expert panel review

To ensure the scientific rigor and practical feasibility of the intervention program, a panel of six experts on mental health and software development was invited to comment on the initial draft of the intervention program. The panel consisted of one psychiatrist, two nursing professionals, two psychotherapists, and one software engineer. Their professional experience ranged from 9 to 29 years. The detailed composition of the expert panel is presented in Table 3.

**Table 3:** Basic information of participating experts (*n* = 6).

Number	Gender	Qualification	Title	Research field	Working years
N1	Female	PhD	Advanced	Psychotherapist	25
N2	Male	M.D	Associate senior	Psychiatrist	13
N3	Female	Undergraduate	Advanced	Psychotherapist	29
N4	Female	Undergraduate	Intermediate	Nursing of mental illness	17
N5	Female	Undergraduate	Intermediate	Nursing of mental illness	19
N6	Male	Postgraduate	Advanced	Software engineer	9

The expert panel meeting was chaired by the principal investigator. Prior to the start of the formal session, experts were informed that the meeting would be audio recorded with their consent, and it was emphasized that the recording would be used solely for research purposes. At the commencement of the meeting, research team members provided a comprehensive overview to the attending experts, covering the background, objectives, anticipated outcomes of the study, and specific details of the intervention plan. Printed copies of the intervention plan were distributed for expert review and discussion. The meeting lasted 90 min.

Following the meeting, research team members collated and summarized the discussion points and key findings, which were then used to revise the study intervention plan. Key revisions following expert consultation included: (i) Adjustment of the age range from 14–60 years to 16–60 years, considering concerns about younger patients' control over mobile phone usage; (ii) Optimization of patient recruitment sources to focus on BD patients who are about to be discharged from psychiatric units, as they have relatively stable conditions and clearer cognition; (iii) Consolidation of psychoeducation themes from eight to six domains for streamlined content delivery; (iv) Establishment of a supervision and management team to monitor intervention implementation and patient completion of functional modules; and (v) Enhancement of privacy protection protocols and reduction of identifiable personal data collection. A detailed account of the topics discussed and the subsequent amendments made by the expert panel is presented in Table 4.

**Table 4:** Discussion and revision content at the expert panel meeting.

Discussion topics	Revisions
1. Whether the scope of intervention targets is appropriate?	**Determination of the age range of intervention patients.** Considering that BD often manifests during adolescence, and patients who receive early intervention have better prognosis than those who receiving late intervention, which is more conducive to improve patients' functional outcomes. And combined previous studies, the age range of the population to be included in this study was initially set at 14–60 years old. After discussion, the experts unanimously agreed that younger patients might have poor control over mobile phone usage, which could lead to other issues. Additionally, their family members might also harbor certain reservations about it, posing certain risks to the implementation of the intervention. Ultimately, it was unanimously agreed to adjust the age range to 16–60 years old. **Determination of the source of intervention patients**. Initially, the patients in this study were planned to be recruited from the outpatient and inpatient departments of the psychiatry department. After discussion, it was noted that early-stage inpatients often have unstable conditions and poor cognitive function. Moreover, some of them may receive electroconvulsive therapy, which can impact their cognitive function. This makes the implementation of the intervention difficult and may interfere with the results. In contrast, patients who are about to be discharged have relatively stable conditions and clearer cognition, meeting the basic requirements for participating in this study. Ultimately, after extensive discussion, the source of patients to be included in this study was optimized to BD patients who are about to be discharged from the outpatient and inpatient departments of the psychiatric and psychological department of a tertiary-A hospital.
2. Ascertaining the optimal intervention timeframe.	**Study duration**. Based on a comprehensive review of previous literature, the duration of similar studies ranges from 2 weeks to 12 months. After discussions with experts, taking into account the time and labor costs of our research team, the duration of this study was ultimately determined to be 24 weeks, consisting of a 12-week intervention period and a 12-week follow-up period. **Intervention indicator evaluation time**. Initially, this study set the intervention period at 12 weeks, followed by a 12-week follow-up. To ensure the proper implementation of the intervention and the integrity of the data, the intervention period was divided into two phases: the first 4 weeks as Phase I of the intervention and the subsequent 8 weeks as Phase II of the intervention. Therefore, the initially planned times for intervention indicator evaluation were at baseline, the 4th week, the 12th week, and the 24th week. After discussions, it was considered that an increase in the number of patient hospital visits would lead to unnecessary expenses for patients. Given that this study mainly involves online interventions and patients can be readily contacted through social media to ensure the follow-up rate, combined with previous research experience, the evaluation times for the final outcome indicators were adjusted to baseline, the 12th week, and the 24th week. The plan for patients to return to the hospital for follow-up at the 4th week was thus canceled.
3. Does the content of psychoeducation align with clinical realities?	**The psychoeducation information** push is a vital component of the intervention strategy in this study, initially comprising eight thematic categories: ① Understanding BD; ② Pharmacological treatment, side effects, and adherence in BD; ③ Disease progression, functional outcomes, and recurrence management; ④ Self-management strategies for patients with BD; ⑤ Prognosis, rehabilitation, and psychosocial influencing factors; ⑥ Family-oriented care, including marriage, childbirth, and legal considerations; ⑦ Policy interpretation and social welfare provisions pertinent to severe mental disorders. Following expert consultation, thematic consolidation was performed, merging themes 2 and 3, as well as themes 4 and 6, to streamline content delivery. Additionally, recognizing the substantial socio-economic burden of BD-classified among six severe mental illnesses, expert input underscored the importance of integrating knowledge of relevant policies and social support systems into the educational framework. Consequently, the finalized thematic structure for psychoeducation dissemination includes six domains: ① Understanding BD; ② Pharmacological treatment, side effects, and adherence; ③ Self-management strategies; ④ Disease prognosis, rehabilitation, and psychosocial factors; ⑤ Family-centered care, focusing on marriage, childbirth, and legal issues; and ⑥ Policy interpretation, legal considerations, and welfare resources related to the management of severe mental illnesses.
4. How to ensure the completion rate of functional modules?	In contrast to previous studies, the adherence and retention rate in online intervention studies are critical. It was unanimously agreed among experts that a supervision and management team should be established to strictly monitor the implementation of interventions and regularly check patients' completion of functional modules. Specifically, following the dissemination of each informational module, team members should promptly communicate relevant content to participants and conduct weekly audits via the back-end system to verify reading status. Participants demonstrating non-compliance should receive immediate follow-up reminders. Furthermore, based on the capabilities of the research infrastructure and assessments by software engineers, automated notifications for medication adherence reporting via the small program are currently infeasible. Consequently, the supervisory team must implement daily reminders to prompt participants to report their medication intake, perform weekly evaluations of reporting compliance through the back-end system, and provide individualized feedback to reinforce adherence and remind them to report as required.
5. Further considerations to be acknowledged	Experts have pointed out that patients with bipolar disorder often exhibit elevated levels of stigma and heightened privacy concerns. Consequently, privacy protection should be prioritized during the development of the small program. Subsequent consultations with software engineers led to a decision to enhance the application's security protocols and to reduce the collection of identifiable personal data, such as mobile numbers and identity information. Additionally, collaborative deliberations among clinical specialists and software developers resulted in a consensus that the intervention strategy proposed in this study demonstrates a certain degree of scientificity and feasibility. It is recommended that further validation be conducted through a pilot study.

## 4 Platform development

### 4.1 Mini-program development

Based on the agile development model (Wilson et al., 2018), the WeChat mini-program and background data management system were iteratively developed and refined. Following this methodology, a WeChat mini-program titled “Mood Calm” (*Chinese name:*心境静) was developed and registered with the Copyright Protection Center of China (*Registered No. 2021SR1280257*). The user-side version of the mini-program, V1.0512 (*compatible with WeChat iOS 8.0.6 and above*), consists of five modules: “Psychological Self-Assessment,” “Monitoring of Physical Activity, Sleep, and Heart Rate,” “Health Information,” “Health Management,” and “Personal Center.” Developed on the WeChat mini-program platform, “Mood Calm” requires no installation and can be assessed directly via WeChat. The healthcare provider-side of the “Mood Calm” mini-program, version V1.0.0 (*optimized for Chrome browser 90.0.4430.212*), includes modules for administrator login, homepage, user management, article management, assessment scale configuration, assessment management, medical order management, follow-up scheduling, nutritional management, and system settings.

**Figure 4:**
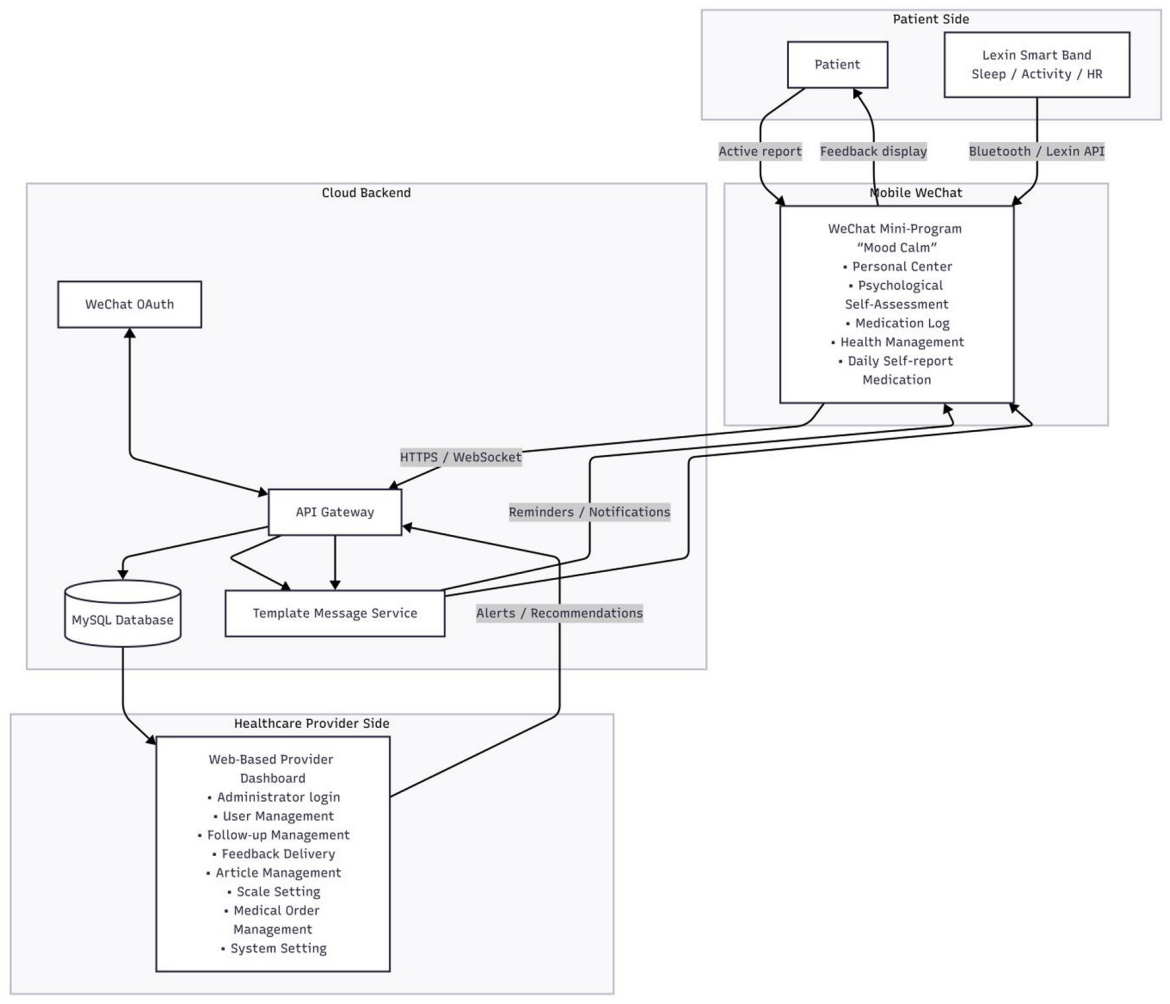
System architecture and data flow of the “Mood Calm” intervention platform. Directed arrows show data flow paths, along with specified protocols and interface details. WeChat OAuth enables secure, registration-free login; MySQL database stores patient metrics and intervention data. The API Gateway orchestrates all data exchanges between components.

Figure 3 shows several screenshots of the “Mood Calm.” Figure 4 illustrates the interaction among four main components: patient side (*user and wearable device*), mobile application (*WeChat* “*Mood Calm*” *mini-program*), cloud backend (*API gateway, MySQL database, WeChat OAuth authentication*, and *template message service*), and healthcare provider side (*web dashboard*).

### 4.2 Pre-launch evaluation

Upon completion of the development phase of “Mood Calm” 1.0, a comprehensive evaluation of the application was conducted by a multidisciplinary team comprising three software engineers, two psychotherapists, four psychiatric nurses, and two psychiatrists prior to initiating the preliminary trial.

## 5 Pilot study

### 5.1 Study design

A preliminary single-arm trial was conducted to assess the feasibility and usability of the “Mood Calm” mini-program. This pilot study employed a pre-post design with a 4-week (*28 days*) intervention period.

### 5.2 Participants and recruitment

A total of five fully or partially remitted patients with BD were recruited from the outpatient clinic of a hospital in Guangzhou City, China, between December 2021 and January 2022. Inclusion criteria for the pilot study were: (i) diagnosed with BD type I or II; (ii) aged 16–60 years; (iii) currently in partial or full remission; and (iv) able to use smartphone application with an active WeChat account.

Eligible patients were informed of the study's purpose and significance by the investigators. After obtaining written informed consent, investigators guided participants to add the “Mood Calm” mini-program and its associated public account, introduced the relevant modules and functions, and collected baseline information (see Figure 3). Since sleep and exercise data need to be collected via the wearable devices (*Lexin Smart Band*), patients are instructed to download the corresponding wristband application (*Lexin Apps*) at enrollment (*the packaging includes a download manual to guide and support patients through the process*). Patients are also informed that it is necessary to open the app once daily to ensure the automatic upload of wristband data, enabling efficient retrieval of relevant information from the back-end system.

Participants completed a 4 week (*28 days*) preliminary trial to assess the usability of the “Mood Calm” mini-program. The sample had a mean age of 19.8 ± 2.32 years, with 80% being female, and all participants possessed at least a high school level education. Table 5 provides a descriptive summary of the pre- and post-intervention characteristics from the preliminary trial.

**Table 5:** Descriptive summary before and after intervention.

Demographic and clinical variables	Pre (*n* = 5) Mean (*SD*)/*n* (%)	Post (*n* = 5) Mean (*SD*)
Age (years)	19.80 (2.59)	–
Gender (female)	4 (80.00)	
Hospitalizations	0.80 (0.84)	–
Age of first onset (years)	16.20 (3.83)	–
Age of first treatment (years)	18.40 (1.82)	–
Chronicity (years)	3.60 (2.41)	–
BMI *(*kg/m^2^)	23.72 (3.60)	25.40 (3.65)
WHR	0.81 (0.07)	0.86 (0.05)
HAMD-17 score	3.60 (1.67)	4.60 (2.30)
YMRS score	2.00 (3.08)	3.00 (2.55)
SAS score	45.40 (8.17)	45.40 (12.46)
SDS score	54.00 (10.56)	50.20 (16.95)
FAST score	8.00 (7.35)	4.20 (1.48)
QoL.BD score	44.20 (2.39)	45.80 (3.03)
SMSs score	106.40 (9.29)	105.20 (10.18)
MCCB score	100.20 (4.09)	102.40 (6.99)
**Sleep**		
Deep sleep time (min)	129.00 (52.37)	135 (68.83)
Light sleep time (min)	317.00 (67.14)	333.00 (55.41)
Total sleep time (min)	446.00 (107.90)	482.00 (72.51)
Awakening time (min)	3.00 (6.71)	11.00 (10.84)
**Exercise**		
Step count (per day)	13,066.20 (6,525.32)	18,251.20 (11,988.25)
Distance (km per week)	29.84 (40.00)	33.54 (43.74)
Calorie consumption (kcal)	1,366.40 (1,726.45)	1,699.40 (2,249.99)
**Biochemical index**		
CHO (mmol/L)	4.63 (0.36)	4.93 (0.48)
TG (mmol/L)	0.91 (0.30)	0.69 (0.14)
HDL (mmol/L)	1.63 (0.29)	1.53 (0.40)
LDL (mmol/L)	2.67 (0.40)	2.79 (0.20)
APOA1 (g/L)	1.52 (0.28)	1.54 (0.13)
APOB (g/L)	0.72 (0.06)	0.74 (0.05)

BMI, Body Mass Index; WHR, Waist-to-Hip Ratio; HAMD-17, Hamilton Depression Scale 17-item; YMRS, Young Mania Rating Scale; SAS, Self-Rating Anxiety Scale; SDS, Self-Rating Depression Scale; FAST, Functioning Assessment Short Test; QoL.BD, Quality of Life in Bipolar Disorder; SMSs, Self-Management Scale; MCCB, MATRICS Consensus Cognitive Battery; CHO, Cholesterol; TG, Triglycerides; HDL, High-Density Lipoprotein Cholesterol; LDL, Low-Density Lipoprotein Cholesterol; APOA1, Apolipoprotein A1; APOB, Apolipoprotein B.

### 5.3 Intervention components tested in the pilot

In the preliminary 4-week trial, only modules that could be delivered entirely through the WeChat mini-program were evaluated. Specifically, the “Mood Calm” mini-program tested the following self-management components: (i) continuous symptom monitoring; (ii) medication management; (iii) maintaining a healthy lifestyle (*sleep and physical activity*) via wearable device integration; and (iv) daily cognitive functioning training.

The psychoeducation component (*six group sessions conducted via videoconferencing*) and the crisis identification and intervention component were not included in this preliminary trial. These components are planned for implementation in a future RCT evaluating the complete intervention. This distinction is methodologically significant. The pilot study evaluated a digital self-management intervention, whereas the future RCT will evaluate a multi-component intervention combining digital self-management with structured psychoeducation.

### 5.4 Operational manuals

The research team developed operational manuals, including the Staff Manual and the Patient User Guide.

**The staff manual** covers: (i) Intervention operation guidelines; (ii) The implementation process of the intervention, including flowcharts and process tables; (iii) The roles and responsibilities of intervention staff; (iv) Detailed implementation procedures, including informational materials, implementation methods, and specific staff assignments; and (v) A software user guide introducing the functions of the “Mood Calm” mini-program, operating environment, software configuration requirements, step-by-step instructions, and troubleshooting measures.

**The patient user guide** includes: (i) Instructions for software access and navigation, including how to launch the “Mood Calm” mini-program, user login procedures, and use of the main modules (*mental health status, step count, sleep, heart rate*, and *health information*), as well as health management features (*medication reporting, medical advice, follow-up plans*, and *nutrition management*); (ii) Descriptions of study participation requirements, including baseline (*Day 1*), Intervention Phase I (*from initiation to Week 4*).

### 5.5 Measures

#### 5.5.1 Feasibility and usability assessment

The feasibility and usability of the intervention were evaluated using a six-item questionnaire designed by the research team. All participants were asked to complete these six questions: (i) Did you find the mini-program easy to use? (ii) Did you experience any lag or technical issues while using the system? (iii) Were there any specific problems you encountered during operation? (iv) Do you think the mini-program assists you in managing your disease? (v) Do you wish to continue using the mini-program for self-management? And (vi) How likely do you think it is that the mini-program will be widely adopted?

#### 5.5.2 Sociodemographic and clinical assessment

All the following data were collected by a clinical interview based on the Structured Clinical Interview for DSM-V (*SCID*). The sociodemographic data included age and gender. Clinical data include number of hospitalizations, age of first onset and treatment, chronicity, body mass index (*BMI*) and waist-to-hip ratio (*WHR*). The YMRS and Hamilton Depression Scale 17-item (*HAMD-17*; Hamilton, 1960) were used to determine the degree of mania and depression by clinical specialists in psychiatry.

#### 5.5.3 Medication adherence assessment

Medication adherence was evaluated using patient-reported medication logs submitted via the “Mood Calm” mini-program. The proportion of days on which medication adherence was self-reported was calculated for each participant.

#### 5.5.4 Emotional symptoms assessment

Anxiety symptoms were assessed using the Self-Rating Anxiety Scale (SAS; Zung, 1971), a 20-item self-report measure. Higher total scores indicate greater anxiety severity. And depressive symptoms also were self-assessed using the 20-item Self-Rating Depression Scale (SDS; Zung, 1965), a 20-item self-report measure.

#### 5.5.5 Functional outcomes assessment

The Functioning Assessment Short Test (*FAST*) is a clinician-administered tool designed to assess psychosocial functioning through 24 items (Rosa et al., 2007). Each item is rated on a four-point Likert scale, ranging from 0 to 3, resulting in a total score from 0 to 72, with higher scores indicating worse functioning. The FAST has been widely used in Chinese patients with BD and has been shown to have good reliability and validity (Zhang et al., 2018).

The Quality of Life in Bipolar Disorder (*QoL.BD*) is a validated self-report tool for assessing quality of life in patients with BD (Michalak and Murray, 2010). Its brief version contains 12 items covering physical, emotional, social, and other life domains. Each item is scored on a 5-point Likert scale, yielding a total score ranging from 12 to 60, with higher scores indicating better quality of life. The Chinese version of the Qol.BD shows strong reliability and validity in Chinese patients with BD (Xiao et al., 2016).

Neurocognitive functioning was assessed using the MATRICS consensus cognitive battery (*MCCB*), which was developed by the US National Institute of Mental Health in 2004. The MCCB is widely applicable for the neurocognitive assessment of BD and other neuropsychiatric conditions. The Chinese normative data for the MCCB demonstrate good validity and reliability, with a Cronbach's alpha of 0.824 (Shi et al., 2015).

#### 5.5.6 Self-management ability assessment

The Self-Management Scale for Bipolar Disorder (*SMS.BD*) was previously developed by our research team (Lai et al., 2022). It comprises 18 items across three dimensions: disease control management, daily life management, and disease knowledge management. It employs a five-point Likert scale, with all items being positively scored. Higher scores indicate better self-management ability. The scale has demonstrated good reliability and validity.

#### 5.5.7 Sleep and physical activity assessment

Sleep and physical activity data were collected using the Lexin Smart Band. Once patients began wearing the bracelets, the devices automatically recorded sleep and activity metrics, which were then converted into their corresponding parameters for storage. The bracelets recorded daily data and uploaded it to the “Mood Calm” mini-program on a weekly basis. Subsequently, weekly averages were computed and used as the principal metrics for evaluating sleep quality and physical activity levels, thereby facilitating a more efficient analysis. In accordance with the literature, the quantification methods were as follows: Sleep quality was evaluated based on four aspects: deep sleep time (*minutes*), light sleep time (*minutes*), total sleep time (*minutes*), and awake time (*minutes*). Physical activity level was evaluated based on the basis of three aspects: the number of steps, distance (*km*), and number of calories burned (*kcal*).

#### 5.5.8 Biochemical index assessment

In the study, a total of six biomarkers were collected, including cholesterol (*CHO*), triglycerides (*TG*), high-density lipoprotein cholesterol (*HDL*), low-density lipoprotein cholesterol (*LDL*), apolipoprotein A1 (*APOA1*), and apolipoprotein B (*APOB*). All individual were taken fasting blood samples about 5 ml at 7:00 a.m. and then placed in a blood collection tube with special measuring instrument by professionals from on hospital. Specific analyses, such as blood centrifugation and serum extraction of these biomarkers, were performed by relevant laboratories.

## 6 Results

### 6.1 Feasibility and usability

**Figure 5:**
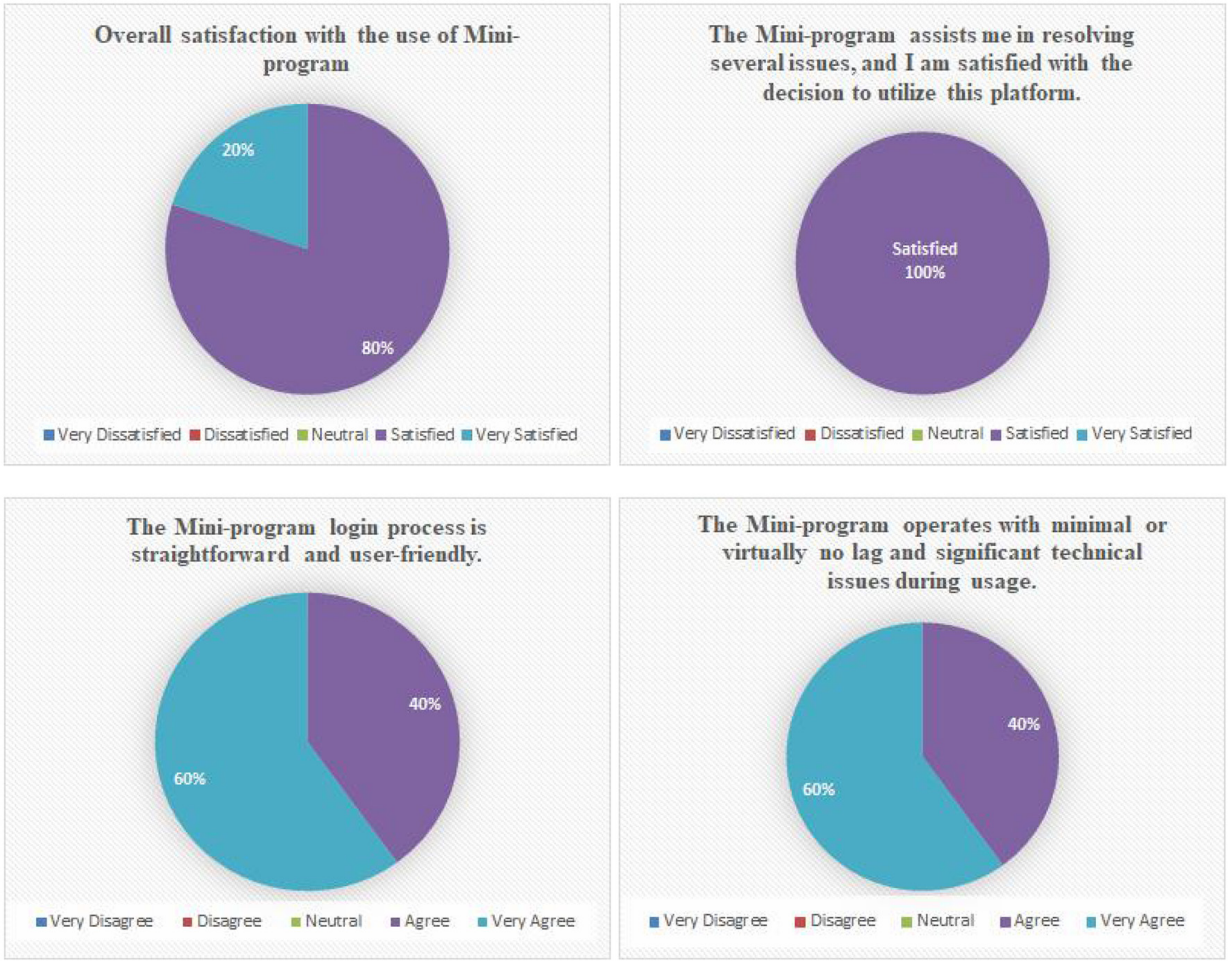
User satisfaction and feasibility of WeChat mini-program “Mood Calm” implementation.

All five patients participating in the preliminary 4-week usability trial successfully completed the self-management intervention. Overall, participants reported high satisfaction with the design and features of the “Mood Calm” mini-program, and unanimously expressed a willingness to recommend it to other patients (see Figure 5). Patients described the program as user-friendly and easy to operate, with no instances of system lag or significant technical issues reported during use (see Figure 5). These findings suggest that the mini-program has strong feasibility and usability, supporting its applications for implementing self-management interventions.

### 6.2 Medication adherence

The proportion of days on which medication adherence was self-reported through the “Mood Calm” mini-program for the five patients was as follows: 26 days (*92.86%*), 15 days (*53.57%*), 14 days (*50.0%*), 15 days (*53.57%*), and 27 days (*96.43%*), respectively. System records indicate suboptimal levels of medication reporting, with only two patients maintaining medication reporting for more than 60% of the intervention days.

### 6.3 Clinical and other variables

Descriptive statistics for clinical, functional, emotional and other variables are presented in Table 5. Given the small sample size and short intervention period, these data are presented for descriptive purposes only and should not be interpreted as efficacy findings. At baseline, mean HAMD-17 scores were 3.60 (*SD* = 1.67) and at post-intervention were 4.60 (*SD* = 2.30). Mean YMRS scores were 2.00 (*SD* = 3.08) at baseline and 3.00 (*SD* = 2.55) at post-intervention. Mean FAST scores showed improvement from 8.00 (*SD* = 7.35) at baseline to 4.20 (*SD* = 1.48) at post-intervention. Sleep data from the wearable device indicated mean total sleep time of 446.00 min (*SD* = 107.90) at baseline and 482.00 min (*SD* = 72.51) at post-intervention. Mean daily step count increased from 13,066.20 steps (*SD* = 6,525.32) at baseline to 18,251.20 steps (*SD* = 11,988.25) at post-intervention.

### 6.4 Participants' suggestions

Participants shared their suggestions during and after the test. Some patients noted that the medication reporting process within the mini-program was cumbersome and easy to forget. As a result, after consultation with the engineering team, the medication reporting module was optimized to streamline the process, enabling one-click reporting and thereby significantly reducing time and improving user compliance. Additionally, during the preliminary trial phase, it was discovered that patients under the age of 18, primarily high school students, faced restrictions in mobile device usage and could not meet inclusion criteria. Since this age group is often in a critical period for college entrance exams and parents are sensitive to mobile phone use, the inclusion criteria for the subsequent RCT were refined to restrict the participant age range 18–60 years, safeguarding the interests of both patients and their guardians.

## 7 Discussion

To the best of our knowledge, this study is the first to assess both the feasibility and usability of a tailored self-management intervention for BD delivered via smartphone technology in China. Our findings indicate that the WeChat “Mood Calm” mini-program may be a feasible and acceptable adjunctive tool for self-management, aligning with the usual treatment for BD. Participants described the application as user-friendly and easy to operate, reinforcing the potential of such digital tools in supporting disease management.

The high satisfaction and positive feedback from our pilot participants are consistent with prior studies reporting good acceptance of smartphone tools for BD self-management (Gliddon et al., 2015; Goulding et al., 2022; Hidalgo-Mazzei et al., 2016; Jonathan et al., 2021; van den Heuvel et al., 2018). However, these results should be interpreted with caution due to the small sample size and short intervention period. Several factors may contribute to this favorable reception. First, the use of a WeChat mini-program eliminates the need for separate downloads and installations, leveraging a platform with exceptionally high penetration in China. This approach directly addresses key barriers to access, such as limited technical literacy and storage constraints, thereby enhancing service availability and reducing entry thresholds (Liu et al., 2023; Ma and Wang, 2022). Second, the integration of a wearable device for passive monitoring of sleep and physical activity aligns with the growing research trend focused on reducing patient self-reporting burden and obtaining objective behavioral data (Cochran et al., 2018; Faurholt-Jepsen et al., 2013). Third, the intervention's content is grounded in established evidence-based components of BD self-management, including symptom monitoring, medication management, crisis identification and intervention, maintenance of a healthy lifestyle, psychoeducation, and daily cognitive functioning training, and is structured within a Cognitive Behavioral Theory framework. This ensures the intervention is both scientifically informed and therapeutically structured, similar to other empirically supported digital interventions (Goulding et al., 2022; Miklowitz et al., 2021). Finally, the development of our intervention protocol followed a rigorous process. The components were derived from a comprehensive literature review, and the preliminary protocol was refined based on feedback from a multidisciplinary expert panel, a practice that enhances content validity. The mini-program was developed using a user-centered design approach in collaboration with software engineers, and its usability was iteratively optimized following a preliminary trial, a methodology emphasized in the development of other digital health interventions (Jonathan et al., 2021).

However, the pilot revealed a notable challenge, that is, suboptimal self-reported medication adherence for three of the five participants. This mirrors a common hurdle in digital health interventions, maintaining consistent long-term engagement (Hidalgo-Mazzei et al., 2016). In response, the research team streamlined the medication reporting process to a one-click entry, exemplifying the advantage of an agile development model that enables rapid iteration based on user feedback (Wilson et al., 2018). This finding underscores that, while technology can facilitate self-management, design must prioritize minimal user effort to support sustained use.

Several limitations of this preliminary trial should be acknowledged. First, the pilot sample was very small (*n* = 5), homogeneous (*young, highly educated*), and evaluated over a short period (*4 weeks*), severely limiting the generalizability of the findings and the ability to assess clinical efficacy. Second, the single-arm, uncontrolled design precludes any conclusions about the intervention's specific effects on symptom severity, relapse rates, or functional outcomes compared to standard care. Third, the pilot evaluated only the mini-program-based components. The psychoeducation and crisis intervention components, which are integral to the complete intervention protocol, were not tested. Therefore, the feasibility and acceptability of the full multi-component intervention remain to be established. Fourth, outcome assessment relied heavily on self-report and system engagement metrics, with the limitation that daily self-reported mania was not captured. Although wearable data were collected, their integration into a closed-loop clinical feedback mechanism requires further development. Fifth, the feasibility assessment was conducted using a non-validated, self-developed questionnaire. Future studies should employ validated usability and acceptability measures to facilitate cross-study comparisons and support more robust evaluations. Finally, the current protocol involves healthcare professionals in monitoring data and issuing reminders. This indicates the model is not purely self-managed and raises questions about the human resource requirements for large-scale implementation.

## 8 Future directions

This pilot establishes a foundation for a randomized controlled trial to rigorously evaluate the efficacy of the “Mood Calm” intervention on key clinical outcomes such as mood stability, psychosocial functioning, quality of life, and medication adherence in a larger, more representative BD population over a longer follow-up period. Future research should also investigate the specific contributions of different intervention components (e.g., *psychoeducation* vs. *passive wearable feedback*) and identify user characteristics predictive of engagement and benefit. Technologically, development could focus on enhancing analytical capabilities, such as implementing algorithms for automated early warning sign detection based on multimodal data (self-report and wearable metrics), enabling more proactive and personalized interventions (Goulding et al., 2022). Furthermore, exploring sustainable models that balance automated support with the necessary human oversight is crucial for scalability.

## 9 Conclusion

In summary, this pilot study provides initial evidence that the WeChat “Mood Calm” mini-program may be a feasible and preliminarily acceptable model for delivering a comprehensive, evidence-based, mini-program-based self-management intervention for BD in China. It leverages a highly accessible platform, combines active and passive monitoring strategies, and is grounded in a theoretical framework. While the pilot results are encouraging regarding usability and acceptability of the mini-program-based components, they do not establish the feasibility of the complete intervention incorporating psychoeducation and crisis management. The study protocol provides a robust foundation for conducting a definitive efficacy trial to determine whether this digitally-supported approach, when combined with structured psychoeducation, can lead to meaningful clinical and functional improvements for individuals living with BD.

## Data Availability

The datasets presented in this study can be found in online repositories. The names of the repository/repositories and accession number(s) can be found in the article/supplementary material.
